# Author’s Postscript: Update on the Guidance of the “Amendments to the Act of the Protection of Personal Information” for Japanese Medical Facilities in May, 2022

**DOI:** 10.2188/jea.JE20220166

**Published:** 2022-09-05

**Authors:** Soichiro Saeki

**Affiliations:** Center Hospital of the National Center for Global Health and Medicine, Tokyo, Japan

In a recent article in the *Journal of Epidemiology*,^1^ the author has noted several possible impacts of the amended Act of the Protection of Personal Information in Japan (APPI), which has been enforced since April 1, 2022. This manuscript corresponds to the previous article in order to update several facts about the APPI, as the Personal Information Protection Commission (PPC) of Japan updated the Q&A for the guideline of the APPI on May 26.^2^

In the previous article, the author pointed out that using patient data obtained in clinical settings may become restricted in many Japanese medical facilities, even in cases where approval has been obtained from the ethical committee to gain opt-out consent from the patients.^1^ However, in the Q&A update on May 26,^2^ the PPC guaranteed the use of clinical information to be used for observational studies, if gaining direct consent from patients without the latest contact information may require significant time and cost, possibly hindering the progress of the study. Although the use of personal information remains limited to its intended use in principle, this guideline could pave the way for some observational studies to be conducted in Japanese medical facilities. In addition, under similar conditions, sharing clinical data among several medical facilities has also been endorsed under strict privacy measures, which could allow multiple-centered observational studies to be conducted as well.

The author fully welcomes this update by the PPC, as this should allow studies that are performed in the same style as previous studies,^3^^,^^4^ which were conducted through a retrospective review of healthcare records. However, the previous discussions regarding to the APPI should serve as a warning to Japanese healthcare professionals that clinical data of patients should not be taken for granted and that personal information of patients must be protected by all means.

The guidance provided by the PPC mentions that studies that have been granted permission should “particularly contribute to the improvement of public health by enabling the results of such research to be widely utilized for the public.”^2^ We, as researchers of medicine, should take this opportunity to bear in our minds once again our mission to contribute to the society through our research, along with the responsibility casted upon us to be fully responsible for the information of our patients.
